# “Women Are Better Than Men”–Public Beliefs on Gender Differences and Other Aspects in Multitasking

**DOI:** 10.1371/journal.pone.0140371

**Published:** 2015-10-19

**Authors:** André J. Szameitat, Yasmin Hamaida, Rebecca S. Tulley, Rahmi Saylik, Pauldy C. J. Otermans

**Affiliations:** Division of Psychology, Department of Life Sciences, Brunel University London, Uxbridge, United Kingdom; University of Tuebingen Medical School, GERMANY

## Abstract

Reports in public media suggest the existence of a stereotype that women are better at multitasking than men. The present online survey aimed at supporting this incidental observation by empirical data. For this, 488 participants from various ethnic backgrounds (US, UK, Germany, the Netherlands, Turkey, and others) filled out a self-developed online-questionnaire. Results showed that overall more than 50% of the participants believed in gender differences in multitasking abilities. Of those who believed in gender differences, a majority of 80% believed that women were better at multitasking. The main reasons for this were believed to be an evolutionary advantage and more multitasking practice in women, mainly due to managing children and household and/or family and job. Findings were consistent across the different countries, thus supporting the existence of a widespread gender stereotype that women are better at multitasking than men. Further questionnaire results provided information about the participants’ self-rated own multitasking abilities, and how they conceived multitasking activities such as childcare, phoning while driving, and office work.

## Introduction

The term multitasking refers to the concurrent performance of at least two tasks. This covers a range of situations, such as the temporally overlapping performance of two or more tasks (e.g. driving while talking on the phone; often also called *dual-tasking*), but also the frequent switching between tasks, even if at a single point in time only one task is performed (e.g. constantly switching between writing emails and answering phone calls; often also called *task switching*).

As researchers in the area of multitasking we made the incidental observation that after giving presentations to the general public, the first question from the audience usually was “Is it true that women are better at multitasking than men?” This pointed to the existence of a general public belief or stereotype which assumes that there are gender differences in multitasking and that these differences are in favour of women. A search in public media such as daily newspapers supported this initial hypothesis and revealed a considerable number of articles and books which depict women to be better at multitasking than men (e.g. [1, 2–6].

However, a frequent problem of such reports in public media is that scientific information is often greatly simplified, reduced, or even misinterpreted in order to support already existing stereotypes. For instance, a recent newspaper article [1] suggested in the title that women are better at multitasking. The newspaper article referred to a study by Ingalhalikar *et al*. [7] in which gender differences in white matter connectivity have been reported. However, Ingalhalikar *et al*. [7] never mentioned the term multitasking and also did not refer to a related concept. In addition, it is unclear whether the observed gender differences in brain connectivity are after all large enough to explain potential real-life gender differences [8]. Thus, it might be that a potential stereotype about gender differences in multitasking is fuelled by suggestive reports in the public media. This is crucial, because it is well known that gender stereotypes can influence the performance of tasks even when their typical performance is independent of gender [9, 10].

But what has previous empirical research revealed regarding gender differences in multitasking? After all, a belief in such gender differences would not be surprising if research would have shown that there are indeed consistent gender differences of an effect size noticeable in everyday life. We split our discussion into two sections, studies which investigated multitasking in terms of rather “low-level” cognitive functions, e.g. in the context of cognitive psychology, and studies which aimed to simulate rather “high-level” real-life tasks, e.g. in the context of applied, work, and/or occupational psychology.

In the first section, we discuss studies from the broad area of cognitive psychology. A rather prototypical multitasking paradigm is the task switching paradigm [11, 12]. In this paradigm participants have to switch between two tasks with ambiguous stimulus- and/or response-sets, e.g. alternating between deciding whether a number between 0 and 9 is either even/odd (task 1) or smaller/larger than 5 (task 2). Typically, constantly alternating between tasks is slower than working on the same task block wise, the so called task-switching costs. Stoet *et al*. [13] have indeed shown gender effects in this paradigm. In more detail, they showed that some task-switching costs are higher for men than for women, i.e. women were better at multitasking. However, it should be noted that these effects were rather small: Males showed higher task-switching costs by just 8%, a difference which just reached statistical significance (p = .03) despite a very large sample size of 240 participants. Another recent study by Mantyla [14], in which a complex self-designed multitask was used, found exactly the opposite, i.e. that men were better at multitasking than women. However, it should be noted that again the effects were rather small: Males showed better accuracy by 10% and a large sample of 72 participants was required to achieve statistical significant results. To our knowledge, there are no other studies in the area of cognitive psychology which investigated gender differences in multitasking directly. However, re-analysis of our own data from the dual-task paradigm of the psychological refractory period did not show any gender differences [15, 16]. In addition, Redick *et al*. [17] reported that in a sample of more than 6000 participants the gender differences observed in complex memory span tasks (which can be considered to be a dual-task) were small to non-existent. Taken together, the evidence from cognitive psychology on gender differences in multitasking is mixed, i.e. sometimes men are better at multitasking and sometimes women are better, but the size of the gender difference is usually very small. In addition, often no differences between men and women are reported at all. Thus, it appears questionable that a potential stereotype on gender differences in multitasking abilities is based on findings from cognitive psychology.

In the second section, we now discuss previous findings from a more applied background. For instance, Watson and Strayer [18] found no gender differences in the ability to drive while phoning. Buser and Peter [19] had participants work on a Sudoku puzzle and a word search task and manipulated whether participants were allowed to freely switch between tasks or not. They showed that there were not only no performance differences between genders, but also that males and females did not differ in their propensity to switch between tasks (i.e., to multitask) either. Finally, Paridon and Kaufmann [20] combined a driving task (lane-change-task) with an office task (determine how many spelling mistakes a visually presented word had) and also failed to observe any gender differences in performance. Given these null-findings it should be noted that from a statistical point of view, a null-finding, i.e. the inability to reject the null-hypothesis, does not allow for accepting the null-hypothesis [21, 22]. However, we would argue that the present null-findings at least indicate that if there truly are gender differences, which just have not been detected due to beta-errors, these effects most likely have rather small effect sizes.

Taken together, the previous evidence on potential gender differences in the performance (i.e. time to finish a task and/or accuracy) of multitasking suggests that there is no clear pattern of gender differences. There are occasional reports showing that women are better than men [13], like there are occasional reports showing the opposite, i.e. that men are better than women [14]. However, most studies that looked for gender differences in multitasking or related paradigms observed no differences between genders (e.g. [17, 18, 19]. To summarize, previous findings suggest that there are no profound and consistent gender differences. Thus, it seems highly unlikely that any potential public belief in gender differences is based on empirical-science based knowledge.

The current study had three main aims. The first aim was to assess how widespread the belief in gender differences in multitasking is at all. So far, there seems to be only some use of this stereotype in the media and our own incidental observations, but it might be that most people actually do not believe in gender differences in multitasking. Therefore, one major part of the questionnaire given to participants focussed on their belief in gender differences, with the additional aim to characterize potential beliefs in more detail (e.g., which gender they believe to be better and why—in particular assessing the role of childcare). Based on the presence of the stereotype in the media and our own incidental observations, we expected that a noticeable proportion of the participants believed that women are better at multitasking than men.

The second aim was to assess how participants judged their own multitasking behaviour (e.g. how many hours per day they thought spend multitasking and how good they are at it), and in particular whether gender differences are evident in these self-judgements.

Finally, we were interested in assessing participant’s conception of multitasking. We did this by presenting participants a list of activities (e.g. Feeding a toddler and talking on the phone; Operating a SAT Nav while driving; Doing paperwork and responding to emails) and asking them how strongly they agree that these activities are examples of multitasking. Following the same logic, we also presented a list of occupations and asked how much multitasking is required for each of them (e.g. Accountant; Housewife/man; Teacher; Office worker).

Besides assessing empirically the extent to which people belief in gender differences in multitasking, we also assessed whether this belief is moderated by certain demographic variables such as gender, age, education, and whether one has children or not.

We implemented the study as an online survey. To assess whether a potential belief in gender differences in multitasking is a more general phenomenon, we distributed the questionnaire in several countries, i.e. the United States, the United Kingdom, Germany, the Netherlands, and Turkey. We would like to note that this should not be considered as a cross-cultural comparison, because we only assured that for each country participants were born and presently living in that country, without taking the cultural background of their parents and grandparents into account. Instead, the wider distribution in several countries serves the sole purpose of getting a first estimate about the generalizability of the observed effects.

## Methods

### Participants

491 participants took part in the study (Table 1). Three cases were excluded due to obvious fake answers, leaving 488 cases (274 females, 212 males) for the analysis. Please note that not all questions were shown to all participants due to conditional logic, and we also analysed incompletely filled out questionnaires, so that the total number of valid responses differs between questions and usually does not add up to 488.

**Table 1:** Participant demographics.

		*Gender*		*Age*		*Children*		*Relationship status*		*Education*	
Country	Total N	Females	Males	< = 35	>35	Yes	No	Single	Married	non-HE	HE
UK	108	62	46	73	35	40	56	63	44	78	30
US	42	38	4	40	2	3	36	5	37	26	16
Germany	57	37	20	27	30	21	31	31	26	32	25
Netherlands	99	58	41	91	8	9	78	37	59	56	41
Turkey	123	38	85	97	28	36	16	53	71	79	44
Other	57	41	16	43	14	13	43	21	36	29	27
**Total (ALL)**	**488**	**274**	**212**	**371**	**117**	**122**	**260**	**210**	**273**	**300**	**183**

*Notes*. N = sample size; HE = highest education is of the category higher education, i.e. usually a university degree or student; non-HE = highest education is high school or below.

The questionnaire was distributed online in the UK (N = 108), US (N = 42), Germany (N = 57), the Netherlands (N = 99), and Turkey (N = 123). Because the questionnaire was distributed online, we also allowed participants from other countries who came across the survey link to participate (“Other”; N = 57). This category of “Other” consisted of a mix of 30 different countries, with the main countries being Morocco (N = 7), Brazil (N = 4), and Bulgaria, Canada, China, Romania, and Switzerland (each N = 3). In the following, we refer to the full sample, i.e. all participants who filled out the respective question, by the term ALL.

Participants were asked to indicate their age in categories: 18–25 years (N = 206); 26–35 years (N = 165); 36–45 years (N = 59); 46–55 years (N = 39); 56–65 years (N = 13), and over 65 years (N = 6). Since the sample sizes decreased considerably with increasing age, for all further analyses we split the participants only into two age groups: younger or equal to 35 years of age (< = 35; N = 371) and older than 35 years (>35; N = 117). We felt that a distinction between < = 35 as “younger” participants and >35 as “older” participants was more appropriate than < = 25 as younger and >25 as older (sample size was too small to make the split at a higher age, or to split into three groups).

Participants were asked about the number of children they have in form of an open question which could have been left blank. 260 participants reported having no children, and 122 participants reported having one or more children (106 participants did not answer this question).

The relationship status was assessed by a number of categories, which were subsumed into the following two main categories: (1) Single (N = 273) and (2) Married (N = 210; including domestic partnerships and civil unions).

Regarding the highest achieved education, participants were categorized into two categories: (1) Higher education (HE, N = 183), including every academic training following high school, such as college, university, being currently enrolled as a university student, PhDs, associate’s degrees and other professional or graduate degrees and (2) all education which stopped at the level of high school or before (non-HE; N = 300).

The study was approved by Brunel University London ethics committee and all participants gave informed written consent before participation.

### Materials

For the purpose of this study, a new questionnaire was developed. The questionnaire started with providing participants some information about the research and asking them to give consent to participate. Next, the demographic questions were presented, which were followed by the main questions. The questionnaire was presented online using SurveyMonkey (SurveyMonkey Inc., Palo Alto, California, USA, www.surveymonkey.com). Except for open comment text box questions, participants had to provide an answer before they could continue. The order of the questions was kept constant across participants. Except for some questions, participants could not see the next question before they answered the current question. Conditional logic was implemented to allow for a meaningful sequence. In particular, if participants stated that they did not believe in gender differences, the questions about which gender is better, why it is better, and how large/significant the differences are were not presented. In addition, the questions about the reasons why people believed in a gender difference were adjusted for each individual gender (e.g. a question was either “Why do you think women are better than men at multitasking?” or “Why do you think men are better than women at multitasking?”, depending on their previous answer to the question whether they think men or women are better). There were no other instances of conditional logic.

The main questionnaire could be subdivided into three topics, which relate to the aims specified in the introduction section: (1) What do participants think of their own multitasking behaviour? (2) Do participants believe there are gender differences in multitasking abilities? If so, questions aiming at characterising the gender differences were presented. (3) What is multitasking?

For the non-English speaking countries the questionnaire was translated into the local language by one of the authors who were native speakers of that language (Germany (AJS), the Netherlands (PCJO), and Turkey (RS)). These authors also translated the open comments made in the respective local language back into English.

The questionnaire was made accessible online using SurveyMonkey from 24th November 2014 to 1st February 2015. It was distributed and advertised via social media, emails, messages, and online forums.

### Data analyses

Data were analysed depending on the scale level. Dichotomous variables (e.g. yes/no answers) were analysed using Chi-square (χ²) tests. For these data, the standard error was estimated as the square root of ((p * (1-p)) / n), and Cramér’s *V* was used to report effect sizes. Responses to scale questions (including 4, 5, or 7 items, depending on the question) were analysed using t-tests. For these data, Cohen’s *d* was used to report effect sizes.

## Results

In the following, the results will be presented question by question in the order they were presented to the participants. Please note that we omit presentation of questions which were not directly related to the research questions of the current manuscript (e.g. “What is multitasking to you?”) or which resulted in only very few answers and could not be analysed properly (some open comment text boxes which were not mandatory to fill out, e.g. “Please share any other opinions you have on the topic of multitasking and gender differences”). Presentation of results will be split by the three main aims of the current study described above.

### Part I: Multitasking abilities

**Fig 1:**
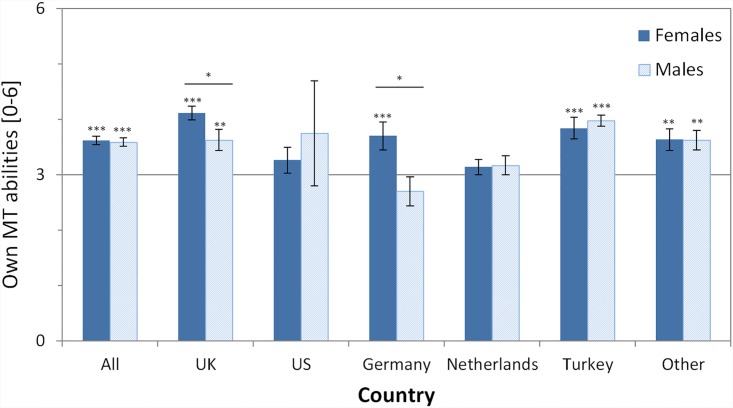
Self-rated own multitasking (MT) abilities as assessed by the question “How good do you think you are at multitasking?”. Scale ranged from 0 (“extremely bad”) to 6 (“excellent”). In total (category ‘All’) 486 participants (274 females; 212 males) answered this question. Results of one-sample t-tests testing whether the mean rating differed significantly from a rating of 3 (“average”; centre of scale) are shown as asterisks above each bar (* p < .05; ** p < .01; *** p < .001). Results of independent samples t-tests testing for gender differences are shown above each pair of bars of a country (* p < .05). Error bars denote standard error of the mean (SEM).

Question 1: We asked the participants “How good do you think you are at multitasking?”, and participants responded on a scale from 0 (“extremely bad”) to 6 (“excellent”). Data showed (Table 2, Fig 1) that for ALL, there was no gender difference in how participants judged their own multitasking abilities (mean ALL females: 3.62, males: 3.59; *t*(486) = .275, *p* = .783, Cohen’s *d* = .025). However, in two country samples, i.e. UK and Germany, women judged their own multitasking abilities higher than males (UK: *t*(106) = 2.18, *p* < .05, *d* = .423; Germany: *t*(55) = 2.56, *p* < .05, *d* = .690). German males were the only group of participants who judged their own multitasking abilities numerically as below average (one-sample t-test vs the mean rating of 3: *t*(19) = 1.14, *p* = .267, *d* = .523).

**Table 2:** Means of the self-rated own multitasking (MT) abilities as assessed by the question “How good do you think you are at multitasking?”.

		Ratings by Gender			Modulated by			
Country	N	Females	Males	*p* (F vs M)	Age	Children	Education	Relations.
UK	108	4.11***	3.63**	0.031*	x	x	x	x
US	42	3.26	3.75	0.532	x	x	x	x
Germany	57	3.70**	2.70	0.013*	x	x	x	x
Netherlands	99	3.14	3.17	0.881	x	x	noHE > HE	x
Turkey	123	3.84***	3.98***	0.492	x	x	x	x
Other	57	3.63**	3.63**	0.978	x	x	x	x
*Total (ALL)*	*486*	*3.62* ***	*3.59* ***	*0.783*	*x*	*C > noC*	*noHE > HE*	*x*

*Legend*. Scale ranged from 0 (“extremely bad”) to 6 (“excellent”). Influences of demographic factors on the mean ratings (averaged across genders) are shown in the right panel (“Modulated by”): statistically non-significant effects are marked by “x”, and statistically significant effects (p < .05) are noted by the direction of the difference. For instance, in the Netherland sample, participants with non-HE background rated their own MT abilities significantly higher than participants with a HE background.

*Notes*. **N** = Number of participants in the sample. ***p* (F vs M)** = p-value of independent samples t-tests comparing females vs males:

* p < .05;

** p < .01;

*** p < .001.

**noHE** = non-Higher Education. **HE** = Higher Education. **C** = having children. **noC** = having no children. **Relations**. = Relationship status.

The self-rated multitasking abilities were affected by a few demographic variables. In particular, for ALL, participants with children (mean rating 3.89) judged their own multitasking abilities to be significantly higher than participants without children (mean rating 3.45) (t(381) = 3.30, p < .005, *d* = .338). In addition, for ALL and the Netherlands, participants with non-higher education (mean 3.74) judged their multitasking abilities to be higher than participants with higher education (mean 3.41) (ALL: t(485) = 3.02, p < .005, *d* = .274). These effects of demographic variables showed largely the same patterns across all individual country samples (although the differences were often not statistically significant), with occasionally countries showing an opposite pattern (but then usually only non-significantly with small effect sizes).

To summarize, there are only weak indications of a gender effect in self-rated own multitasking abilities. In two countries (UK and Germany), women judge themselves significantly better than males, but this is not a consistent pattern across all samples and consequently fails to reach significance for ALL. Participants with children and non-higher education judge their own multitasking abilities to be higher than the respective other samples.

**Fig 2:**
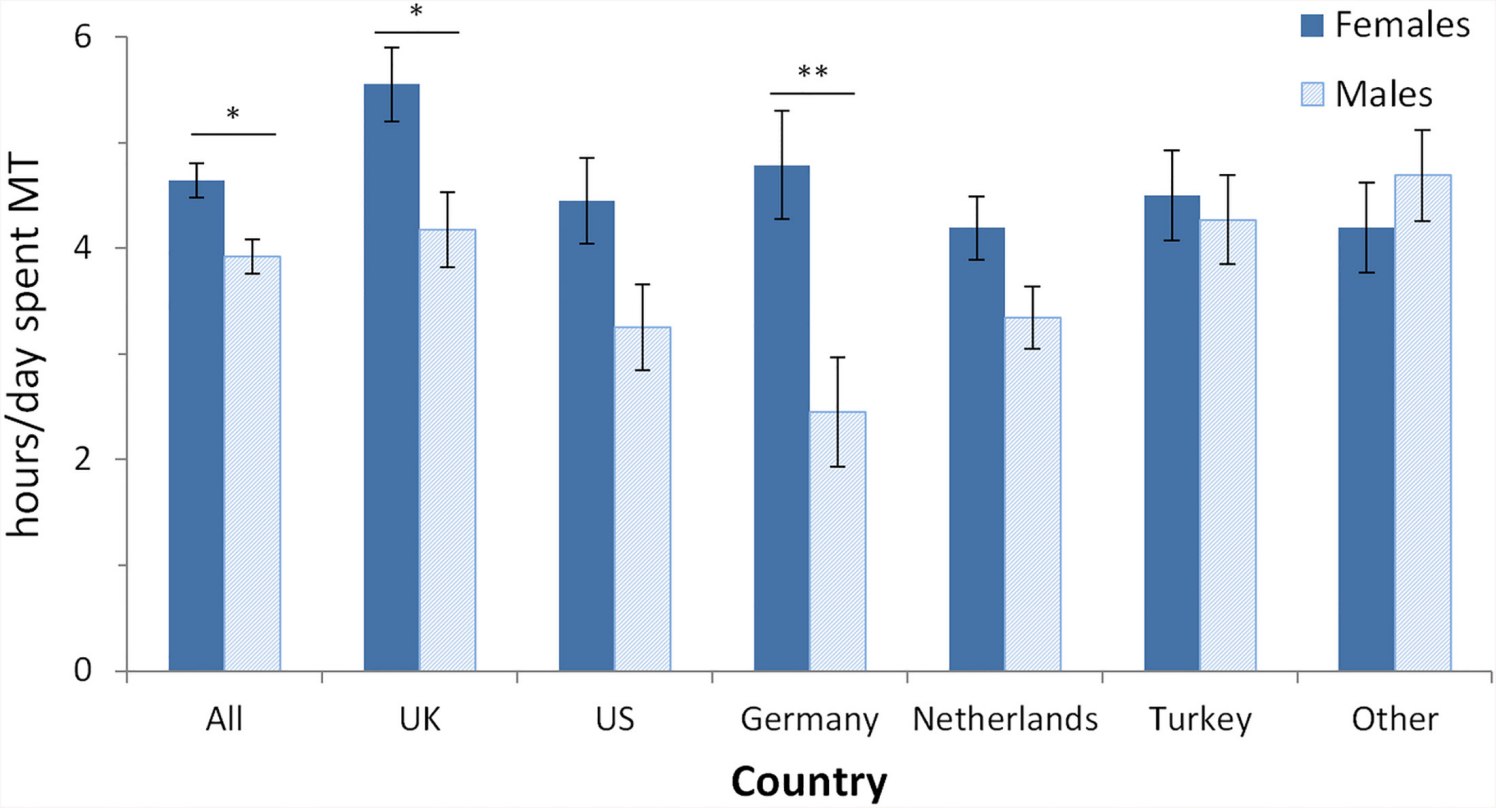
Means of the self-rated hours spent multitasking (MT) per day. In total (category ‘All’) 486 participants (274 females; 212 males) answered this question. Results of independent samples t-tests testing for gender differences are shown above each pair of bars of a country (* p < .05; ** p < .01). Error bars denote SEM.

Question 2: We asked participants “How many hours a day do you spend multitasking?” Participants answered by selecting one of the provided categories, which was recoded for parametric analyses (possible answer category [recoded value]: 0 [0]; 1 [1]; 2–4 [3]; 5–7 [6]; 8–10 [9]; +10 [11]). Results (Table 3, Fig 2) showed a strong gender effect. For ALL, females (mean 4.64 hours/day) estimated to spend on average 43 minutes more on multitasking than males (mean 3.92) (t(486) = 3.00, p < .005, *d* = .272). The same pattern was evident for all country samples, except Other.

**Table 3:** Means of the self-rated hours spent multitasking (MT) per day.

		Ratings by Gender			Modulated by			
Country	N	Females	Males	p (F vs M)	Age	Children	Education	Relations.
UK	108	5.55	4.17	0.015*	x	C > noC	x	M > S
US	42	4.45	3.25	0.358	x	x	x	x
Germany	57	4.78	2.45	0.004**	x	x	x	x
Netherlands	99	4.19	3.34	0.062	x	x	x	x
Turkey	123	4.50	4.27	0.646	x	x	x	x
Other	57	4.20	4.69	0.557	x	C > noC	noHE > HE	x
*Total (ALL)*	*486*	*4.64*	*3.92*	0.003**	*x*	*C > noC*	*noHE > HE*	*M > S*

*Notes*. **M** = married, domestic/civil partnership, divorced, widowed. **S** = single, never married.

The self-rated hours spent multitasking were affected by several demographic variables. Participants (for ALL) with children (mean 5.13 hours/day) reported to spend 1 hour per day more multitasking than participants without children (mean 4.13), (t(381) = 3.43, p < .001, *d* = .351). Participants (for ALL) with a non-higher education (mean 4.54 hours) reported to spend 30 min more multitasking per day than participants with higher education (mean 4.04), (t(483) = 1.99, p < .05, *d* = .181). Finally, for ALL, married participants (including domestic / civil partnerships, and formerly married participants, e.g. divorced, separated, and widowed) (mean 4.66 hours) reported to spend 33 min more with multitasking per day than participants who reported to be single (mean 4.11), (t(483) = 2.27, p < .05, *d* = .207). Again, these effects of demographic variables showed largely the same patterns across all individual country samples, even if often failing to reach statistical significance.

To summarize, there are strong effects of the self-rated hours the participants spend multitasking each day. In particular, females report to spend 45 min more multitasking per day than males, parents (i.e. participants with children) 60 min more per day than non-parents, non-higher educated 30 min more than higher educated, and married 33 min more than singles (all numbers refer to ALL). We would like to point out that these are self-reported hours of multitasking, and not objectively assessed hours spent multitasking. Thus, the present findings cannot decide whether e.g. women just think that they multitask more, or whether they indeed actually do multitask more.

### Part II: The belief in gender differences

**Fig 3:**
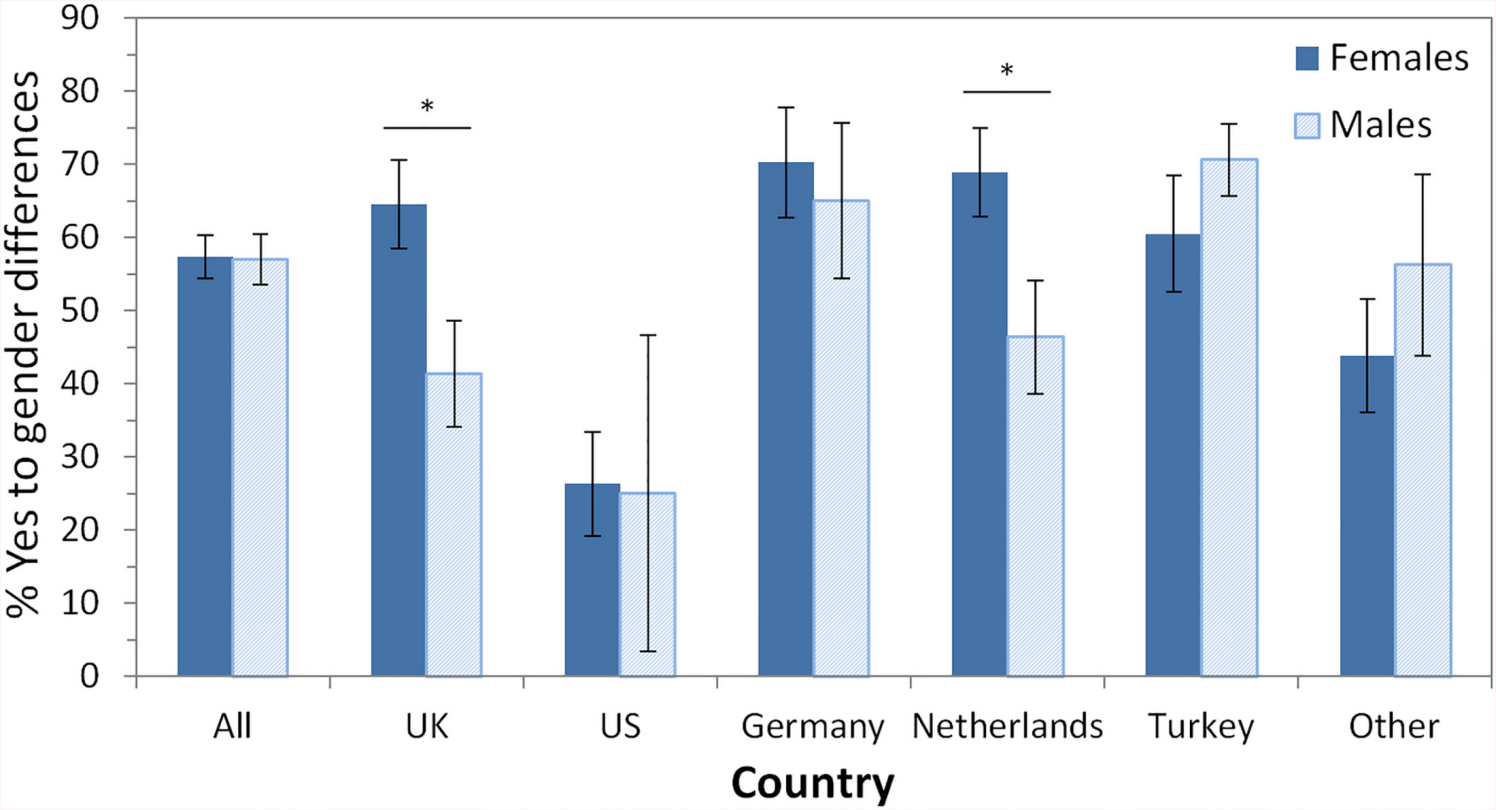
Proportion “Yes”-responses (in percent, %) in response to the question “Do you think there are gender differences in multitasking?”. Dichotomic scale with Yes and No as answer options. In total (category ‘All’) 486 participants (274 females; 212 males) answered this question. Results of χ² tests testing for gender differences are shown above each pair of bars of a country (* p < .05). Error bars denote standard error.

Question 3: We asked the participants “Do you think there are gender differences in multitasking?” with the answer options “Yes” and “No”. At this stage, participants were not able to preview any further questions. Results presented in Table 4 and Fig 3 depict the proportion of participants who believed in gender differences, i.e. the ones who answered Yes to this question. For ALL, 57% of all participants believe in gender differences. To estimate the statistical significance of the finding, we calculated the 95% confidence interval and report the lower boundary of it in Table 4. For ALL, this is 52%, which means that at least 52% of the participants believe in gender differences (with p < .05).

**Table 4:** Proportion “Yes”-responses (in percent, %) in response to the question “Do you think there are gender differences in multitasking?”. Dichotomic scale with Yes and No as answer options.

		Overall Ratings		Ratings by Gender			Modulated by			
Country	N	Yes (%)	Lower bound CI (%)	FemalesYes (%)	MalesYes (%)	p (F vs M)	Age	Children	Education	Relations.
UK	108	55	45	65	41	0.017*	x	x	x	x
US	42	26	15	26	25	0.955	x	x	x	x
Germany	57	68	55	70	65	0.683	Y > O	x	x	x
Netherlands	99	60	49	69	46	0.024*	x	x	x	x
Turkey	123	67	58	61	71	0.271	x	x	x	x
Other	57	47	34	44	56	0.402	Y > O	x	x	x
*Total (ALL)*	*486*	*57*	*52*	*57*	*57*	*0.931*	*x*	*x*	*x*	*M > S*

*Notes*. “Overall Ratings” = mean of all respondents (i.e., an average of Female and Male ratings weighted by male and female sample sizes). “Lower bound CI” = Lower bound of the 95% confidence interval. It reflects the maximum value below the observed percentage which shows a significant difference (p < .05; determined using X^2^ tests). For instance, 57% of All participants believe in gender differences, which is significantly more than 52%. **Y** = Younger participants (35 years or younger). **O** = Older participants (36 years or older).

The belief in gender differences in multitasking was affected only by few demographic variables. For ALL, 10% more married participants (63%) believed in gender differences than single participants (53%). This was statistically tested by a χ² test based on a 2x2 table (Belief in gender differences [Yes/No] x relationship status [married/single]), which confirmed the significance of the difference (χ²(1) = 4.52, p < .05, Cramér’s *V* = .097). Though significant only for ALL, all countries except the Netherlands showed the same pattern. In Germany and Other, younger people (< = 35 years) believed significantly more in gender differences than older people (>35 years), but other countries showed the opposite effect, so that there is no clear overall pattern evident.

To summarize, overall more than half of the participants (ALL: 57%) believe in gender differences in multitasking. For the different countries this varied between only one in four participants believing in gender differences (US: 26%), up to two in three participants believing in gender differences (Germany: 68%). While it is clear that not all participants do believe in gender differences, we conclude that substantial amounts of our sample do. In other words, we found support for the existence of gender stereotypes in multitasking.

**Fig 4:**
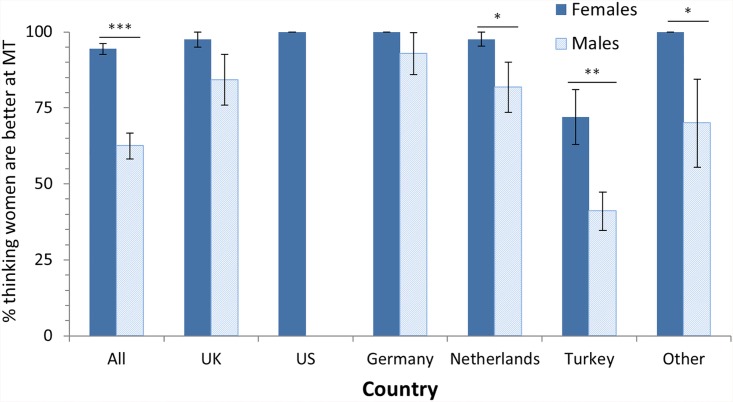
Proportion (in percent, %) of participants choosing women in response to the question “Who do you think is better at multitasking?”. Dichotomic scale with Men and Women as answer options. In total (category ‘All’) 288 participants (160 females; 128 males) answered this question. Results of χ² tests testing for gender differences are shown above each pair of bars of a country (* p < .05; ** p < .01; *** p < .001). This question was only presented to participants who believe in gender differences (Question 3). Error bars denote standard error.

Question 4: We asked the participants “Who do you think is better at multitasking?” with “men” and “women” as answering options. This question was presented only to those participants who believed in gender differences, i.e. the ones who answered *Yes* to Question 3. Results in Table 5 and Fig 4 depict the percentage of participants answering that women are better at multitasking. Since there were only two answer options, the percentage of participants thinking that men are better can be derived by subtraction the women-percentage from 100 (e.g. for ALL, 80% of the participants thought women were better, i.e. 20% thought men were better at multitasking).

**Table 5:** Proportion (in percent, %) of participants choosing women in response to the question “Who do you think is better at multitasking?”.

		Overall Ratings	Ratings by Gender			Modulated by			
Country	N	*“Women”* (%)	Females *“Women”* (%)	Males *“Women”* (%)	p (F vs M)	Age	Children	Education	Relations.
UK	59	93	98	84	0.058	x	x	x	x
US	11	91	---	---	---	---	---	---	---
Germany	40	98	100	93	0.168	x	x	x	x
Netherlands	64	92	98	82	0.025*	x	x	x	x
Turkey	86	51	72	41	0.009**	x	x	x	x
Other	28	89	100	70	0.014*	x	x	x	x
*Total (ALL)*	*288*	*80*	*94*	*63*	0.001***	*x*	*noC > C*	*x*	*x*

*Legend*. Dichotomic scale with Men and Women as answer options (therefore, the proportion of participants who chose Men can be calculated by 100-%Women). This question was only presented to participants who believe in gender differences (Question 3).

*Notes*. Please note that “Overall” is the percentage of participants choosing women across the whole sample of male and female participants. Since the numbers of males and females are usually not the same, “Overall” is not just the mean of the “Female” and “Male” columns, but a weighted average. Because the US sample is too small for this question (N = 11; 10f/1m), no tests were calculated.

As can be seen in Table 5 (column “Overall”), a large majority of participants (80%) who believe in gender differences do believe that women are better at multitasking, except for Turkey. If Turkey, which showed a different pattern, is not considered, then even 93% of the remaining participants believe that women are better (varying between Other 89% and Germany 98%). If this is related to the total sample size (again excluding Turkey) including the participants who do not believe in gender differences, then 188 out of 363 participants (52%) believe that women are better at multitasking (including Turkey: 232 out of 486 participants, i.e. 48%). In Turkey this believe is also present, but less pronounced, with a gender effect in that 72% of the females believe women are better and 59% of the males believe men are better at multitasking. In other words, in Turkey participants think their own gender is better at multitasking.

The belief that women are better at multitasking was affected by the gender of the participants: Generally, more females (ALL: 94%) believe that women are better, while less males (63%) do believe so (ALL: χ²(1) = 45.9 p < .001, *V* = .399). In addition, the effects were affected by whether participants had children or not. Participants without children thought much more frequently (ALL: 92%) that women are better than participants with children (ALL: 77%), (ALL: χ²(1) = 8.81, p < .005, *V* = .201).

To summarize, among the people who believe in gender differences, there is an overwhelming majority who believe women are better. In particular, virtually all female participants think women are better, while the percentage of male participants thinking women are better is significantly lower, but still very high. An exception to this pattern is Turkey where participants think their own gender is better at multitasking. Finally, more non-parents believe women are better than parents. Thus, this question confirmed the existence of a gender stereotype in multitasking, and further refined it, showing that indeed most people believe that women are better at multitasking.

Since the absolute number of participants believing men are better than women is very low, we conducted no further analyses for that group.

Question 5: We asked the participants “Why do you think they are better?” This question was presented only to the 288 participants who believed in gender differences (Question 3). It was an open text box question, which could be skipped by participants without giving an answer. Individual comments were categorized into common generalized reasons. Only participants who believed women are better at multitasking chose to give comments, and 208 participants made a comment. If participants specified more than one reason, each reason was counted individually, so that overall 223 reasons were given (Table 6). Demographic variables were not considered for this question.

**Table 6:** Summarized answers to the open comment question “Why do you think they are better?”.

*Reasons for why women are better at multitasking than men*	*N (%)*
Due to the demands of concurrently managing household and children, and/or family and job	33 (16%)
Due to differences in the brain, genetics, evolutionary development, instinct, and/or natural predisposition	33 (16%)
Own experience and observation (that women are good and/or men are poor at multitasking)	31 (15%)
Because women have more practice and/or experience in multitasking, they have a greater need for it	26 (13%)
Because women are better in focussing on two things at once, have better concentration abilities, and/or can better split their attention	24 (12%)
Because it is common sense, common knowledge, and/or a stereotype	15 (7%)
Because women are better at organising, planning, and/or scheduling	14 (7%)
Because women are taught to do so during childhood development and/or due to societal pressure during development	10 (5%)
Because women work more / have more responsibilities and therefore need multitasking to manage the workload	8 (4%)
Women get less stressed by multitasking and/or are more patient	7 (3%)
Women can faster switch between tasks	5 (2%)
Other (e.g. higher intelligence (N = 3); are faster (N = 2); shown by research (N = 2))	17 (8%)

*Notes*. Percentages rounded; Percentages relative to number of participants (208) and not to sum of statements (i.e., percentages add up to > 100%).

To summarize, this question revealed a few main reasons why participants thought women are better at multitasking. There are two main themes among the most frequent answers: (1) the belief that women have a natural predisposition for multitasking, e.g. due to evolutionary development and/or differences in brain functions and/or genetics. (2) The notion that women need more multitasking than men, e.g. because of the demands to balance childcare and household chores, or to balance family and work. This might lead to increased practice and therefore improved multitasking abilities.

Another frequent comment was that participants felt it is their own experience that women are better. Comments in this category were given well balanced by males and females alike, either females noting that they seem to manage multitasking situations more effortlessly as compared to close friends, spouses, colleagues, etc., or males noting that they seem to perform worse than females close to them.

**Fig 5:**
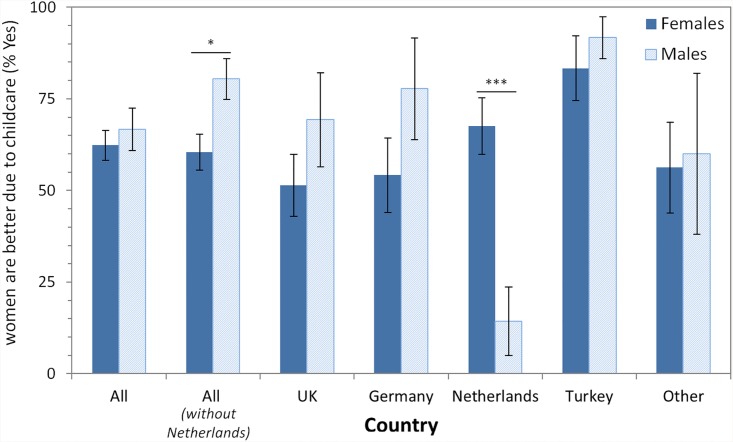
Proportion (in percent, %) of participants answering “Yes” in response to the question “Do you think women are better due to childcare? For example having to attend to children’s needs as well as other household tasks.”. Dichotomic scale with Yes and No as answer options. In total (category ‘All’) 199 participants (136 females; 63 males) answered this question. Because the Netherlands’ sample showed a very strong and opposite pattern as compared to all other samples, we present a further category which subsumes all participants across all countries except for the Netherlands sample (“All (without Netherlands)”). Results of χ² tests testing for gender differences are shown above each pair of bars of a country (* p < .05; ** p < .01; *** p < .001). Error bars denote standard error.

Question 6: We asked the participants “Do you think women are better due to childcare? For example having to attend to children’s needs as well as other household tasks.” with possible answers being “Yes” and “No”. We explicitly asked for this because in our informal observations this was given as the main reason why people approaching us believed women were better at multitasking. Results (Table 7, Fig 5) show that for ALL, 64% of all participants who believed women are better at multitasking believed that this is due to childcare. Overall, this effect was not modulated by demographic variables.

**Table 7:** Proportion (in percent, %) of participants answering “Yes” in response to the question “Do you think women are better due to childcare?”.

		Overall Rating		Ratings by Gender		
Country	N	Yes (%)	Lower bound CI (%)	Females	Males	p (F vs M)
UK	48	56	42	51	69	0.269
US	8	75	40	---	---	---
Germany	33	61	43	54	78	0.216
Netherlands	51	53	39	68	14	0.001***
Turkey	38	88	75	83	92	0.409
Other	21	57	36	56	60	0.882
*Total (ALL)*	*199*	*64*	*57*	*62*	*67*	*0.546*

*Legend*. Dichotomic scale with Yes and No as answer options. Effects were not modulated by age, children, education, or relationship status.

*Notes*. “Overall Ratings” = mean of all respondents (i.e., an average of Female and Male ratings weighted by male and female sample sizes).

A closer inspection of the data revealed that there were two opposing effects of the gender of the participants. In most countries, more males than females thought that women are better due to childcare. However, in the Netherlands a very strong and highly significant opposite effect was present, i.e. much more females than males thought women are better due to childcare (χ²(1) = 11.6, p < .001, *V* = .477). Thus, for this question we calculated a new ALL variable which summarizes all countries except the sample from the Netherlands. This analysis showed indeed a gender effect: More males (80%) than females (60%) thought that women are better due to childcare (χ²(1) = 6.14, p < .05, *V* = .201).

To summarize, overall two in three participants who believed that women are better at multitasking do believe that women are better at it due to childcare. Generally, more men than women believe that childcare is the reason, with the exception of the Netherlands were more women than men think that childcare is the reason.

Question 7: We asked the participants “Do you think women who don’t have children are also better at multitasking (e.g. because they have a genetic / evolutionary advantage)?” with the possible answers “Yes” and “No”. We asked this question to better understand why childcare might be considered a factor in having better multitasking abilities. This question was only presented to those participants who believed that childcare is the reason why women are better at multitasking.

Results (Table 8) show that a considerable percentage of participants (ALL: 73%) believed that even women without children are better at multitasking than men. This suggests that most participants believe women’s advantage is not a pure practice effect acquired since birth, but instead is an inherent, possibly evolutionary advantage.

**Table 8:** Proportion (in percent, %) of participants answering “Yes” in response to the question “Do you think women who don’t have children are also better at multitasking (e.g. because they have a genetic / evolutionary advantage)?”.

Country	N	Yes (%)	Lower bound CI (%)
UK	21	48	28
US	2	---	---
Germany	13	92	66
Netherlands	27	78	59
Turkey	38	87	72
Other	9	33	12
*Total (ALL)*	*111*	*73*	*64*

*Legend*. Dichotomic scale with Yes and No as answer options. Because sample sizes are small, no detailed analyses were performed (For ALL, there were no modulations by gender, age, children, education, or relationship status).

**Fig 6:**
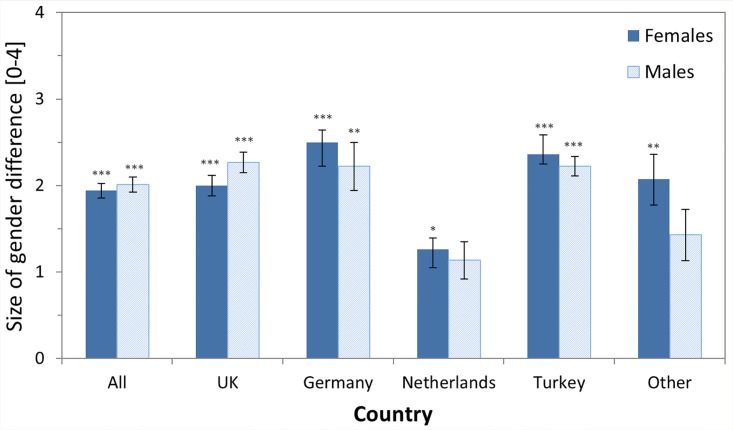
Means of the rated size of the gender differences as assessed by the question “How large do you think the [gender] difference is?”. Scale ranged from 0 (“very little”) to 4 (“significant”). In total (category ALL) 238 participants (136 females; 102 males) answered this question. Results of one-sample t-tests testing whether the mean rating differed significantly from a rating of 1 (“Little difference”) are shown as asterisks above each bar (* p < .05; ** p < .01; *** p < .001). Error bars denote SEM.

Question 8: We asked the participants “How large do you think the difference is?” with five possible answers ranging from “Very little difference” (recoded as 0) to “Significant difference” (recoded as 4). This question was presented to all participants who believed in gender differences (i.e. answered “Yes” in Question 3). Results (Table 9, Fig 6) showed that on average, participants rated the size of the gender differences roughly to be “moderate” (rating 2, i.e. middle of the scale). To assess the statistical significance of this finding, we compared the mean ratings versus a value of 1 (“little difference”) using one-sample t-tests, separate for each country and gender. This analysis revealed that in all countries, the female participants’ ratings were significantly higher than 1 (“little difference”), while it was significant for males only in ALL, UK, Germany, and Turkey (ALL females: t(136) = 11.76, p < .001, *d* = 2.02; ALL males: t(101) = 11.68, p < .001, *d* = 2.324). For ALL (and quite a few further samples), the mean rated difference in gender effects was even significantly larger than 1.5 (i.e., halfway between little and moderate difference) (ALL females: t(136) = 5.5, p < .001, *d* = .943; ALL males: t(101) = 5.90, p < .001, *d* = 1.174). There were no significant differences between male and female participants.

**Table 9:** Means of the rated size of the gender differences as assessed by the question “How large do you think the [gender] difference is?”.

		Ratings by Gender			Modulated by			
Country	N	Females	Males	p (F vs M)	Age	Children	Education	Relations.
UK	49	2.00***	2.27***	0.182	x	x	x	M > S
US	8	---	---	---	---	---	---	---
Germany	31	2.50***	2.22**	0.338	x	x	x	x
Netherlands	53	1.26*	1.13	0.599	x	x	x	x
Turkey	76	2.36***	2.22***	0.538	x	x	x	x
Other	21	2.07**	1.43	0.177	x	x	x	x
*Total (ALL)*	*238*	*1.94* ***	*2.01* ***	*0.567*	*x*	*C > noC*	*x*	*x*

Scale ranged from 0 (“very little”) to 4 (“significant”). Results of one-sample t-tests testing whether the mean rating differed significantly from a rating of 1 (“Little difference”) are shown as asterisks:

* p < .05;

** p < .01;

*** p < .001.

The rating of how large the gender differences in multitasking abilities are was affected by only two demographic variables. For ALL, participants with children (mean 2.13) thought the difference is larger than participants without children (mean 1.85) (t(178) = 2.04, p < .05, *d* = .306). In addition, in the UK, married participants (mean 2.05) thought the difference is larger than single participants (mean 1.90) (t(46) = 2.67, p < .05, *d* = .787). However, both effects showed some variability across the different samples so that a generalization of the findings should be done with caution.

To summarize, participants think that gender differences in multitasking abilities are of a moderate size. This excludes the possibility that participants may think that there is a difference, but that this difference is negligible. Instead, they think that the gender difference is surely more than just a “little” difference. While this question focussed on the size of the difference, the next question focussed on the everyday relevance of the gender difference.

**Fig 7:**
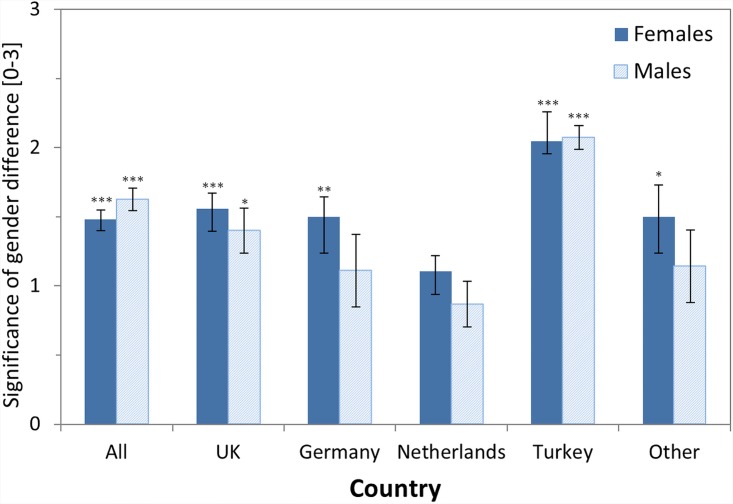
Means of the rated significance of the gender differences as assessed by the question “How significant/relevant for everyday life do you think the [gender] difference is?”. Scale ranged from 0 (“not relevant”) to 3 (“very relevant”). In total (category ‘All’) 238 participants (136 females; 102 males) answered this question. Results of one-sample t-tests testing whether the mean rating differed significantly from a rating of 1 (“slightly relevant”) are shown as asterisks above each bar (* p < .05; ** p < .01; *** p < .001). Error bars denote SEM.

Question 9: We asked the participants “How significant/relevant for everyday life do you think the difference is?” with four possible answers ranging from “Not relevant” (recoded as 0) to “Very relevant” (recoded as 3). This question aimed at assessing whether participants believed that there is any everyday relevance to the presumed gender differences. It was presented to all participants who believed in gender differences (i.e. answered “Yes” in Question 3). Results (Table 10, Fig 7) showed that the mean rated everyday significance is roughly halfway between “Slightly relevant” (rating of 1) and “Relevant” (rating of 2). To test this finding statistically, we compared the mean ratings, separate for each gender and country, versus a value of 1 (i.e., “slightly relevant”). This analysis showed, that for all countries (except the Netherlands which was n.s.) all female participants and most male participants assign an everyday relevance of gender differences in multitasking, which is significantly higher than “slightly relevant” (ALL females: t(136) = 7.00, p < .001, *d* = 1.200; ALL males: t(101) = 7.73, p < .001, *d* = 1.538). Note that all genders and countries assigned a rating significantly higher than 0 (“Not relevant”). There were no differences between female and male participants.

**Table 10:** Means of the rated significance of the gender differences as assessed by the question “How significant/relevant for everyday life do you think the [gender] difference is?”.

		Rating by Gender			Modulated by			
Country	N	Females	Males	p (F vs M)	Age	Children	Education	Relations.
UK	49	1.56***	1.40*	0.436	x	x	x	x
US	8	---	---	---	---	---	---	---
Germany	31	1.50**	1.11	0.174	x	C > noC	x	M > S
Netherlands	53	1.11	0.87	0.253	x	x	x	x
Turkey	76	2.05***	2.07***	0.882	x	x	noHE > HE	x
Other	21	1.50*	1.14	0.351	x	x	x	x
*Total (ALL)*	*238*	*1.48*	*1.63*	*0.171*	*x*	*C > noC*	*noHE > HE*	*x*

*Legend*. Scale ranged from 0 (“not relevant”) to 3 (“very relevant”). Results of one-sample t-tests testing whether the mean rating differed significantly from a rating of 1 (“slightly relevant”) are shown as asterisks:

* p < .05;

** p < .01;

*** p < .001.

The rating of the everyday significance of gender differences was affected by a few demographic variables. Participants with children (mean 1.71) assigned a significantly higher everyday significance than participants without children (mean 1.32) (ALL: t(178) = 3.33, p < .005, *d* = .499). In addition, non-higher educated (mean 1.65) assigned a higher everyday significance than higher educated (mean 1.38) (ALL: t(236) = 2.51; p < .05, *d* = .327). These effects were rather stable across all samples.

To summarize, participants do not only think that the size of the difference is more than a “little”, they also think that the gender difference is more than “slightly” relevant for everyday life. Parents and non-higher educated participants assign a higher everyday significance of the gender differences than non-parents and higher educated participants.

**Fig 8:**
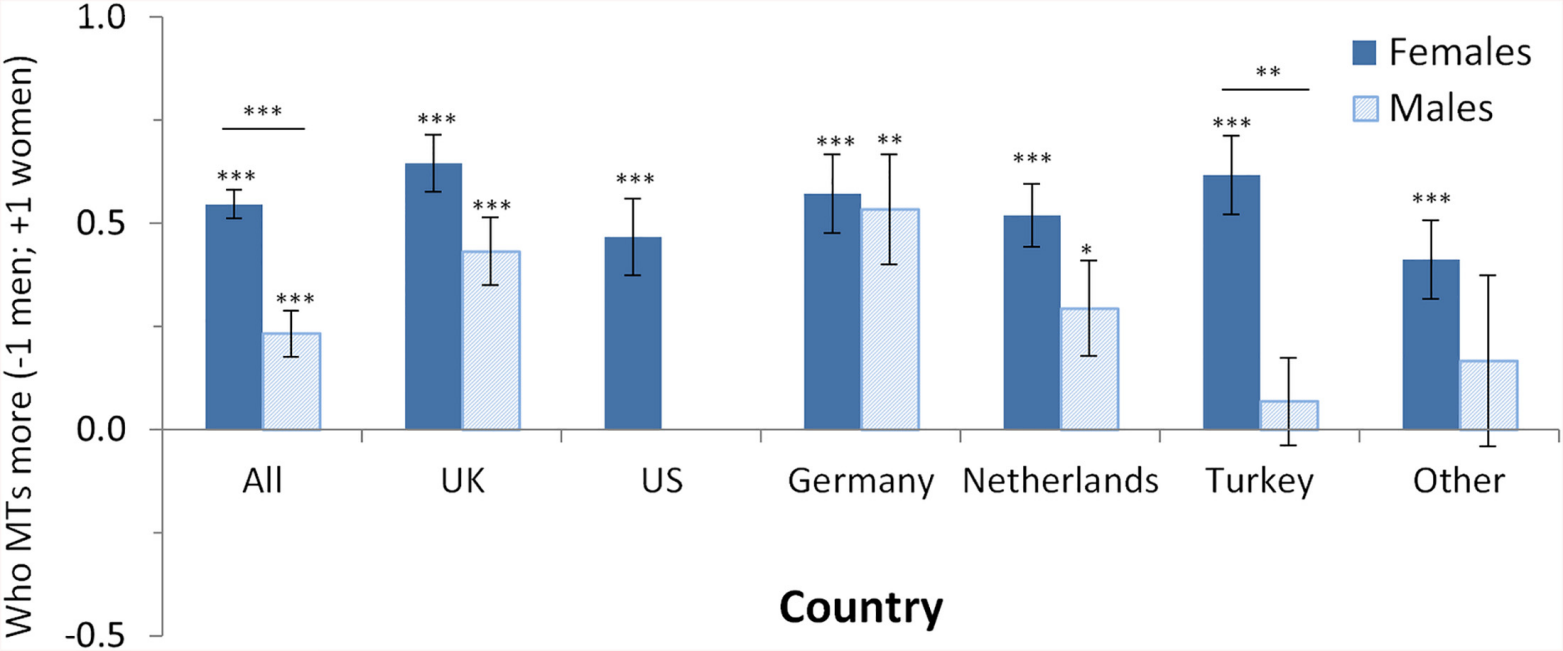
Means of the answers to the question “Who do you think multitasks more?”. Possible answers were “Men” (recoded as -1), “Same” (recoded as 0) and “Women” (recoded as 1). Thus, a positive value reflects that participants think women multitask more. Results of one-sample t-tests testing whether the mean rating differed significantly from a rating of 0 (“same”) are shown as asterisks above each individual bar (* p < .05; ** p < .01; *** p < .001). Results of independent samples t-tests testing for gender differences are shown above each pair of bars of a country (* p < .05; ** p < .01; *** p < .001). Error bars denote SEM. For further details, see Fig 1.

Question 10: We asked the participants “Who do you think multitasks more?” with the answer options “Men” (recoded as -1), “Women” (recoded as 1), and “They spend the same amount of time multitasking.” (recoded as 0). This question aimed at understanding potential beliefs in gender differences in more detail, because it did not ask about abilities or whether one gender is “better” than the other, but rather neutrally about the amount of time spent multitasking. The question was given to all participants, i.e. those who believed in gender differences as well as those who didn’t believe in gender differences. For the analyses, we calculated arithmetic means for each country and gender-subgroup based on the recoded values, so that positive values reflect that participants think women multitask more (these parametric analyses were confirmed by non-parametric χ² tests). Results (Table 11, Fig 8) showed that there is a strong belief that women spend more time multitasking for female and male participants (one-sample t-test vs 0 (i.e., both spend the same amount of time multitasking) for ALL-females: t(224) = 15.6, p < .001, *d* = 2.08; ALL-males: t(175) = 4.14, p < .001, *d* = .626). More female participants (mean .55) thought that women spend more time than male participants (mean .23) (ALL: t(399) = 4.94, p < .001, *d* = .495). Demographic variables had no effect.

**Table 11:** Means of the answers to the question “Who do you think multitasks more?”.

		Rating by Gender		
Country	N	Females	Males	p (F vs M)
UK	85	0.65***	0.43***	0.051
US	34	0.47***	0.00	0.079
Germany	43	0.57***	0.53**	0.816
Netherlands	84	0.52***	0.29*	0.094
Turkey	107	0.62***	0.07	0.001**
Other	46	0.41***	0.17	0.231
*Total (ALL)*	*401*	*0.55*	*0.23*	*0.001* ***

*Legend*. Possible answers were “Men” (recoded as -1), “Same” (recoded as 0) and “Women” (recoded as 1). Thus, a positive value reflects that participants think women multitask more. Results of one-sample t-tests testing whether the mean rating differed significantly from a rating of 0 (“same”) are shown as asterisks:

* p < .05;

** p < .01;

*** p < .001.

Effects were not modulated by age, children, education, or relationship status.

To summarize, the opinion across all participants, including those believing in gender differences as well as those not believing in it, is that women actually do spend more time multitasking.

### Part III: Conception of multitasking

**Fig 9:**
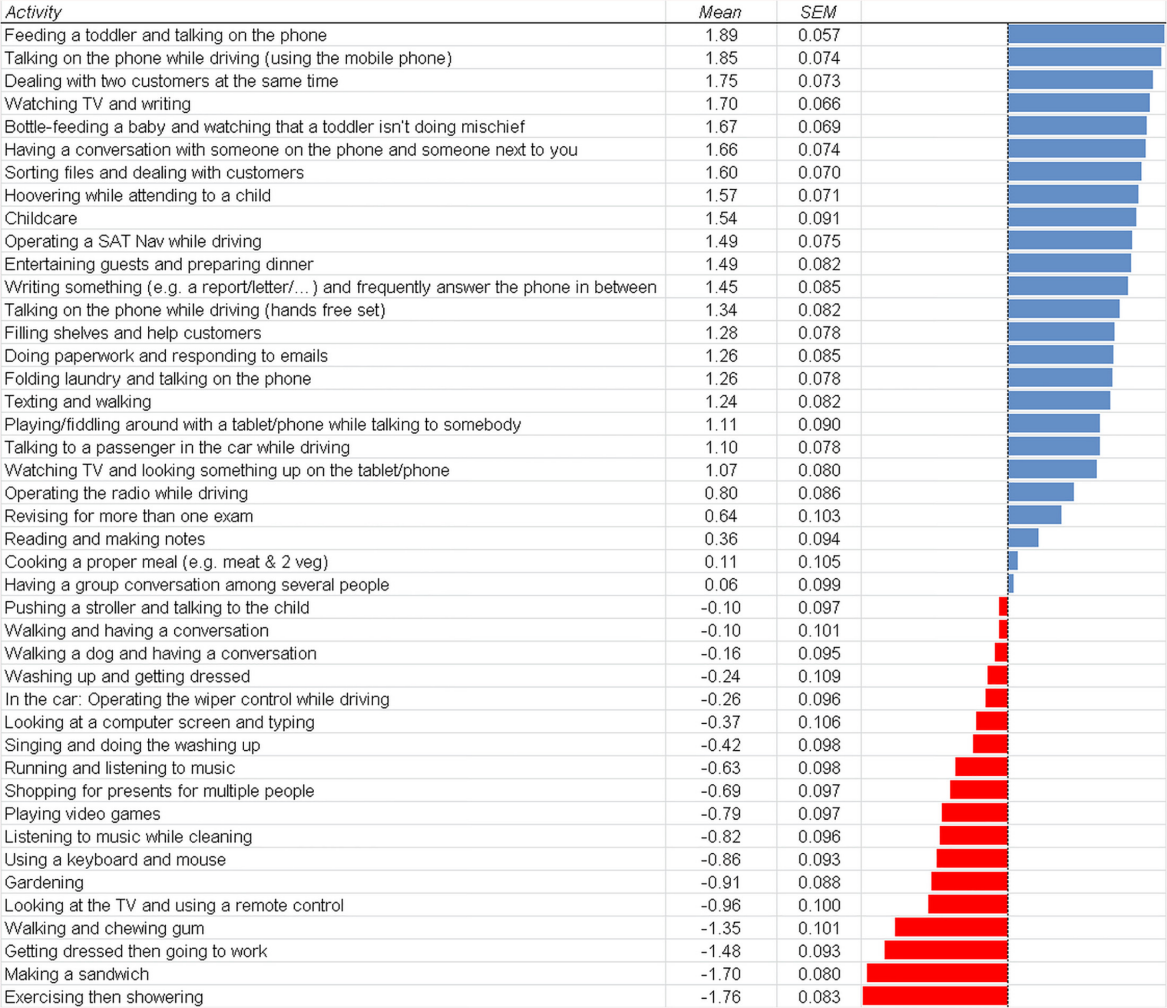
Means of the answers to the question “How far do you agree with the following being examples of multitasking?”. Possible answers ranged from “Strongly disagree” (recoded as -3) to “Strongly agree” (recoded as +3). SEM = standard error of the mean.

Question 11: In the next section we presented participants with 43 activities and asked them “How far do you agree with the following being examples of multitasking?” with seven possible answer options ranging from “Strongly disagree” (recoded as -3) via “Neutral” (recoded as 0) to “Strongly agree” (recoded as 3). Results are shown in Fig 9. For a detailed description and analysis of the results see the Discussion section.

**Fig 10:**
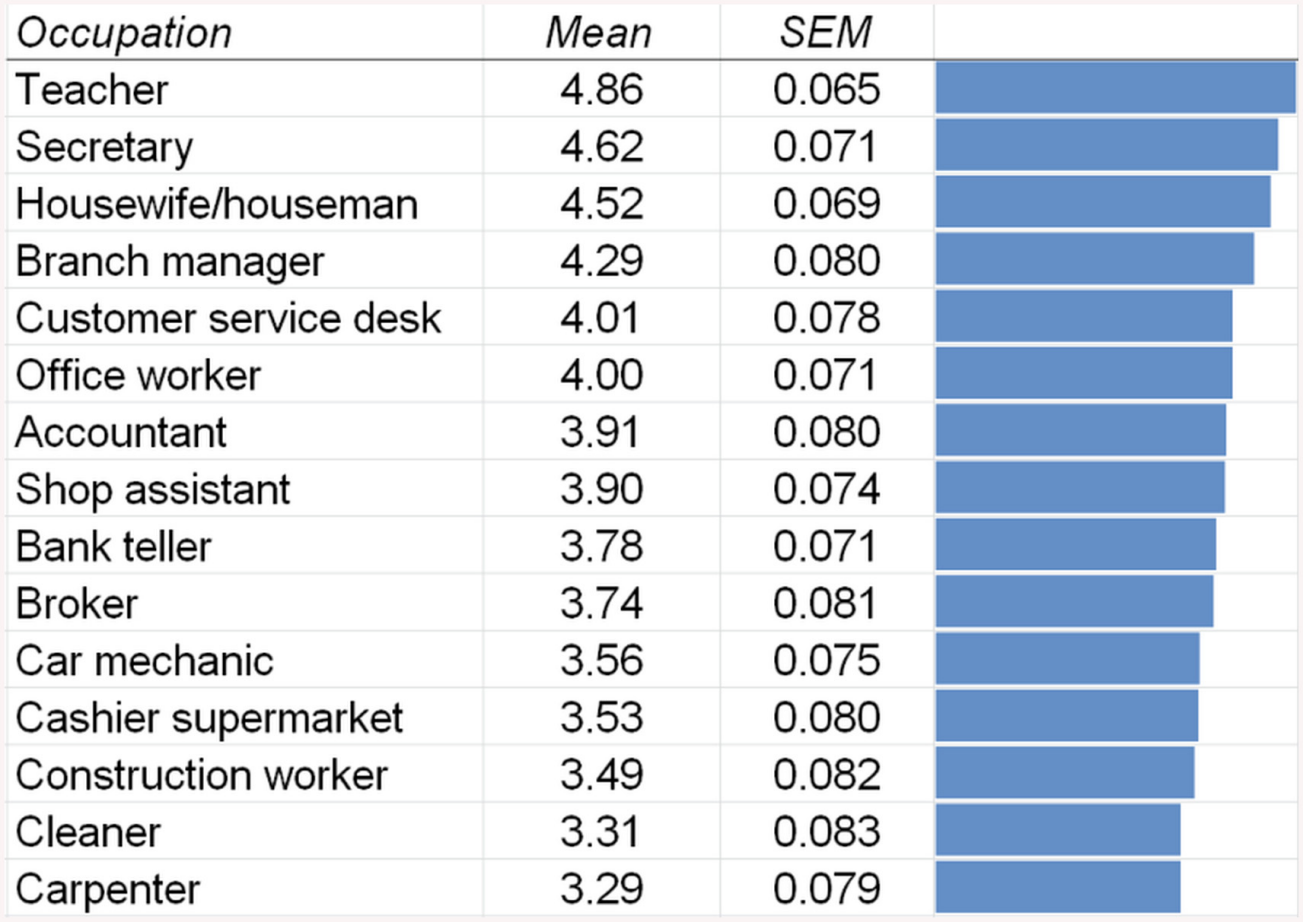
Means of the answers to the question “What do you think, how much multitasking is required in the following occupations?”. Possible answers ranged from “Not at all” (recoded as -3) to “A great deal” (recoded as +3). SEM = standard error of the mean.

Question 12: In the next section we presented participants 15 occupations and asked them “What do you think, how much multitasking is required in the following occupations?” with seven possible answer options ranging from “Not at all” (recoded as 0) via “Somewhat” (recoded as 3) to “A great deal” (recoded as 6). Results are shown in Fig 10. For a detailed description and analysis of the results see the Discussion section.

## Discussion

### Summary of findings

The main aim of this study was to assess how widespread a potential belief in gender differences in multitasking is. We have shown that across all country samples more than half of the participants believed in gender differences. Of those people who did believe in gender differences, the majority (80%) believed that women are better at multitasking. Thus, we were able to show that there is indeed a profound belief that women are better at multitasking than men. Further questions aimed at characterising this belief showed that participants believed women are better due to the concurrent demands of managing children and household (or family and job), due to inborn differences in e.g. brain organisation, and due to more experience, practice, and need for multitasking. The fact that people believe women have an inborn advantage is illustrated by the fact that 73% of the participants thinking women are better due to childcare think that even women without children are still better at multitasking than men. The finding that participants believed that women are *better* at multitasking (i.e., ability) is accompanied by the finding that (male and female) participants also strongly believed that women actually *do spend* more time multitasking than men (i.e., experience). Finally, participants think that the gender difference has a notable size and is relevant for everyday life.

The second aim of this study was to assess how participants judge their own multitasking behaviour. With respect to their own multitasking abilities, there was—interestingly—no difference between male and female participants. In other words, the very same sample of participants who showed a strong belief in gender differences in multitasking abilities did not show any gender difference when asked to judge their own abilities. However, females reported to spend 45 minutes more multitasking per day than males.

Finally, the third aim of this study was to assess the participant’s conception of multitasking. Results showed that mainly the temporally overlapping performance of two demanding tasks was considered as the most accurate examples of multitasking, while the sequential performance of two tasks was not considered to be multitasking as much. Participants also rated teacher to be the occupation (among the presented ones) with the highest demands on multitasking, followed by secretary and housewife/man. The least multitasking was assigned to cleaner and carpenter.

### “Women are better than men”–The belief in gender differences in multitasking

The present data strongly suggest the existence of a gender stereotype: 50% of all participants and, more specifically, 80% of the participants believing in gender differences, believe that women are better at multitasking than men. This finding is in agreement with the many non-scientific newspaper articles and popular books propagating a female advantage in multitasking (e.g. [1, 2, 6, 23, 24]. Note that the present data cannot answer what came first: Whether there has been a gender stereotype in the general public which has been picked up by the media, or whether the media were (for some reason) initiating and spreading this stereotype before it was adopted by the general public. The present study confirms, however, that a substantial stereotype is present today.

Some of the media’s claims are based on theories such as the hunter-gatherer hypothesis [25], which proposes that the division of work led to differences in task performance through natural selection. The idea is that males, whose most important role was to hunt for food, have excelled at an action system tailored towards spatial proficiency and fast perception-action coupling (see also [7]. On the other hand, females, whose most important role was to raise the offspring, prepare food, and engage in more general “housekeeping” activities, have excelled at an action system which enables them to manage these concurrent demands. However, to our knowledge, there is no scientific proof that these potentially different roles indeed led to an evolutionary multitasking advantage in women.

It is interesting to note that also the participants in the present study who believed that women are better at multitasking often used the above arguments. In particular, 64% of participants believed that women are better at multitasking because of the concurrent demands of household and childcare. Following this up, 73% of those participants believed that also women without children are better at multitasking, indicating that participants believe that the multitasking advantage of women is caused by inborn (i.e., evolutionary) factors, and not by a pure practice effect.

### Stereotype, true difference, or both?

Generally, stereotypes like the currently observed one can arise without any true differences to support it, as already noted by Gordon Allport [26]: *“It is possible for a stereotype to grow in defiance of all evidence*.*”* (p. 189). On the other hand, it has been argued that stereotypes often actually correctly reflect some truly underlying differences [27]. Therefore, the question arises whether the belief in multitasking gender differences is a reflection of actual differences in multitasking abilities or, as discussed in the introduction, whether it is an inaccurate belief in regards to empirical research. Although some previous empirical research has demonstrated gender differences in such abilities, they are usually inconsistent and small. Therefore, previous empirical research suggests that the stereotype is non-evidence based.

However, there are some further conceivable explanations. Firstly, it is possible that true gender differences do exist, but that empirical research failed to observe them so far, e.g. because not the right paradigms and tasks have been used for investigations. Generally, there are only a rather limited number of studies looking at gender differences in multitasking abilities, so that this explanation seems conceivable. Alternatively, it might be that participants confused or equated the amount of time spend multitasking and the proficiency in multitasking. Our own study showed that women reported to multitask more, and female as well as male participants thought that women do spend more time multitasking. This is supported by previous research indicating that in everyday life indeed women seem to engage more in multitasking [28]. However, in our study participants were well able to differentiate between these two alternatives, as is illustrated by the presence of a gender difference in self-rated time spend multitasking but the absence of gender differences in the self-rated multitasking abilities. Further research might be required to finally resolve this question.

Taken together, based on absence of any known sizeable gender effects in multitasking abilities, we conclude that at the present time it seems that the belief in gender differences in multitasking abilities is a pure stereotype without supporting empirical evidence.

### Participants’ conception of multitasking

This section discusses the results from Questions 11 and 12, i.e. where participants were asked to rate different situations and occupations. Generally, it seems that participants from the general public classify situations as being a good example of multitasking if at least two main criteria are fulfilled. The first criterion seems to be that the situation involves more than one task at the same time. For instance, the situations with the highest ratings are situations with truly concurrent task performance (i.e. dual-tasking such as phoning while driving), while the situations where people would switch between tasks but only perform one task at one time (i.e. task switching such as doing paperwork and responding to emails) are rated somewhat lower as good examples for multitasking. Finally, the examples where two separate and independent actions follow each other only after their completion (e.g. “Exercising then showering”) are not considered to be multitasking.

The second criterion seems to be that the concurrent performance of the two tasks is very likely to be demanding (in terms of requiring effort/attention) and/or results in performance decrements. This refers to the observation that situations where one or both tasks are very simplistic or even automatic are considered not to be good examples of multitasking, even if the two tasks are temporally fully overlapping (e.g. Looking at the TV and using the remote control; Using a keyboard and mouse; Listening to music while cleaning; etc.).

There are some noteworthy findings which will be discussed in the following. Firstly, the demands of attending to children and doing household chores are among the situations most highly rated as being good examples of multitasking. This is in agreement with the many participants who believed that women are better at multitasking because of the concurrent demands of childcare and household and that women often just have more practice in multitasking (i.e., more need for it).

Secondly, it is interesting to note how the different activities when driving a car were rated. The highest multitasking demands (the overall second highest) were attributed to phoning while driving when using the mobile phone itself. Somewhat surprisingly, this was rated even higher than operating a SAT Nav while driving, which usually requires much more user interaction than taking or making a phone call. Notably, the third highest demanding activity in the car was phoning while driving when using a hands-free set. This is supported by Strayer and Johnston [29] who showed that physically holding the phone actually contributes only little to the attentional demands and the consequential deterioration in driving abilities of phoning while driving. It suggests that people are aware of the problems imposed by phoning while driving, including hands-free set phone calls. Participants only slightly agreed to the suggestions that talking to other passengers in the car or operating the radio while driving are good examples of multitasking, indicating that these actions are considered less demanding and disruptive. Finally, operating the wiper control while driving was not considered to be a good example of multitasking. Overall, this trend nicely illustrates the above notion of the two main criteria for multitasking: The need of two concurrent tasks which are complex enough to make the situation demanding to deal with. As soon as at least one of the tasks becomes too simplistic or automatic (e.g. operating the radio or wiper control), the situations are much less conceived to reflect multitasking.

After the multitasking situations, we presented the participants with a few occupations and asked them how much multitasking would be required for them. We expected that the more managerial occupations (e.g. branch manager) would receive the highest ratings, but our findings were somewhat different. The most multitasking demands were assigned to the job of teacher, followed by secretary. Ranked third was housewife/houseman, even ahead of the branch manager. The lowest multitasking demands were assigned to carpenter, cleaner, and construction worker. However, even for those occupations, an above-average rating of at least 3.29 (of the 0–6 scale with 3 being the central item) was assigned. This illustrates that participants believe that nowadays most occupations involve at least some amount of multitasking. We did not ask participants to explain their views so that the present study cannot identify the reasons why certain occupations are believed to involve more multitasking than others.

### Generalizability of findings

Overall, the data revealed a surprising consistency across the different country samples. Furthermore, demographic variables usually modulated the overall patterns only slightly, but usually did not result for instance in opposite result patterns. One of the few exceptions is the Turkish sample: While more than 50% of the females and males of all countries believe that women are better at multitasking, 59% of Turkish males believe that men are actually better at it. However, we would like to point out that the current study is not aimed at identifying cross-cultural differences. For instance, among other limitations, we assigned a participant to the country which was specified as birthplace and current residence. If they were not the same, the participant was assigned to the group of “Other”. However, we did not assess the cultural background of the parents and grandparents to ensure that the participant was solidly embedded in the assumed cultural background of that country. Nevertheless we would like to argue that the wide spread of countries across North America, Europe, and Western Asia (Turkey) and the overall consistency of findings in these countries suggest that the currently observed gender stereotype is a rather general phenomenon.

### Limitations

By splitting the sample according to demographic subgroups (e.g. by gender, age, etc.) we occasionally created sub-samples with rather unequal sample sizes. In some circumstances, this might result in a statistical bias increasing the likelihood of a beta-error (i.e., missing a true effect). However, please note that this does not affect the main analyses regarding the overall belief in gender differences.

In all countries except Turkey, more females than males participated. This is in agreement with previous studies which identified generally higher response rates in females [30, 31]. Why in Turkey more males than females replied is unclear, but may be related to differences in computer use, a cultural difference that in Turkey females show lower response rates (cf. [32], or a random sampling effect specific to the present study.

The individual country samples usually had different numbers of males and females. In Tables 4, 5, and 7, we report an “Overall Rating” across all participants. We would like to point out that this data is the average of all individual responses, and therefore it is weighted by the gender-specific sample sizes. For instance, if there are more females in a sample than males, then the Overall Rating does reflect proportionally more strongly the female ratings. For an Overall Rating which evenly weights males and females, one can just calculate the mean of the individual male and female ratings as specified in the mentioned tables.

Online surveys generally suffer from a selection bias because only people with access to a computer can participate. In the present study, selection bias might have been further introduced by the mode of recruitment. In particular, we posted invitations to participate in the survey in a number of online forums / bulletin boards targeting different audiences (e.g. car forums, computer forums, childcare forums, horseback riding forums, pensioners’ forums, etc.). We aimed at mainstream forums to reach as many potential participants as possible. Thus, people who browse the internet but have interests not covered by our distribution efforts could not participate. However, since the online recruitment was conducted by different researchers for each country, there were differences in the exact type of forums used, so that the presence of a strong bias across all country samples is unlikely.

It is conceivable that further variables not assessed by us might influence the responses of the participants, e.g. whether participants replied in response to a personal email invitation, a somewhat personal social media post, or a rather impersonal forum post. Unfortunately, we do not have data to conduct such analyses. However, an analyses of the weekday (Monday-Sunday) and time of day (morning, afternoon, evening, night) showed only very minor influences (<5% on average) on the responses on our two main questions (Question 3: Are there gender differences? and Question 4: Who is better?). In combination with the overall very minor effects of the demographic variables assessed by us suggests that the present results are a rather general phenomenon across the population.

### Conclusion

Taken together, we found strong evidence supporting a gender stereotype in multitasking abilities: A considerable proportion of participants from a variety of countries believe that women are better at multitasking than men. This stereotype exists despite the absence of strong empirical data which would justify such a belief. Further research might resolve whether there truly are no gender differences in multitasking abilities or whether previous research just did not look at the right tasks and paradigms.

## Supporting Information

S1 Table Raw data table of the collected data. The three datasets excluded due to obvious fake answers are not included due to the use of strong language in open comments textboxes. They are available on request from the authors.
(XLSX)
